# The Differential Role of Coping, Physical Activity, and Mindfulness in College Student Adjustment

**DOI:** 10.3389/fpsyg.2020.01858

**Published:** 2020-08-04

**Authors:** Robert W. Moeller, Martin Seehuus, Jack Simonds, Eleanor Lorton, Terumi Smith Randle, Cecilia Richter, Virginia Peisch

**Affiliations:** ^1^Department of Psychology, Middlebury College, Middlebury, VT, United States; ^2^Department of Psychological Science, College of Arts and Sciences, The University of Vermont, Burlington, VT, United States

**Keywords:** mental health, college students, stress, coping, mindfulness, physical activity

## Abstract

Research has examined the function of stress management techniques, including coping, physical activity, and mindfulness on college students’ adjustment. The present study examined the differential contributions of three stress management techniques to students’ maladaptation (perceived stress, depression, anxiety, and loneliness) and adaptation (self-esteem). Undergraduate students (*N* = 1185) responded to an online survey. Hierarchical linear regression results indicated that all three stress management techniques – coping, physical activity, and mindfulness – were related to the five outcomes as predicted. Higher levels of disengagement coping strategies were related to higher perceived stress, anxiety, and depression. Components of mindfulness emerged as a strong predictor of adaptation.

## Introduction

### College Student Stress

In line with prior research documenting a link between perceived stress and negative health outcomes (McMahon et al., 2003; Eiland and Romeo, 2013; McCormick and Green, 2013), students transitioning to college have reported increased problems across several domains (Conley et al., 2014). This includes mental health symptoms. One study found that nearly half (45.8%) of surveyed college students experienced a psychiatric disorder, personality disorder, or substance use disorder in the past 12 months (Blanco et al., 2008). Focusing specifically on depressive symptoms Selkie et al. (2015) reported a point prevalence rate of 17% for major depressive disorder in college students. Further, about 6–10% of college students experienced suicidal ideation in the past 12 months (Wilcox et al., 2010; Eisenberg et al., 2013).

Taken together, research suggests that many college students are experiencing significant physical and psychological problems that appear to be associated with elevated levels of perceived stress. Several approaches for the management of stress have been examined, including coping, physical activity, and mindfulness; each of these stress management approaches will be briefly discussed in the following sections.

### Stress and Coping

Coping can be broadly defined as any behavior intended to manage stress (for definition and discussion, see Lazarus and Folkman, 1984). Prior research has identified significant individual differences in the kinds of emotional, physiological, and cognitive coping responses deployed in response to a variety of life stressors (Skinner et al., 2003; Carver and Connor-Smith, 2010). Such individual differences in coping methods can partially explain why people experience stress in vastly different ways (Connor-Smith and Compas, 2004). Some broader classification schemes of coping include problem-focused versus emotion-focused coping (Folkman and Lazarus, 1980), approach versus avoidance coping (Roth and Cohen, 1986), and engagement versus disengagement coping (Compas et al., 2001). Focusing on the classification scheme of engagement and disengagement coping (Carver and Connor-Smith, 2010), research generally suggests that the former is associated with adaptive functioning whereas the latter is associated with maladaptation (see, for example, Compas et al., 2001; Clarke, 2006; Carver and Connor-Smith, 2010; see Villatte et al., 2015 for a discussion of the clinical implication of avoidance). Examples of engagement coping include problem-solving, support-seeking, and positive thinking; disengagement coping includes avoidance, denial, and wishful thinking (Compas et al., 2001).

### Stress and Physical Activity

Although physical activity has been associated with reductions in perceived stress and anxiety and an elevation of overall mood (Dunn et al., 2001; Penedo and Dahn, 2005; Galper et al., 2006; VanKim and Nelson, 2013), levels of physical activity generally decline across the transition from adolescence to young-adulthood (Butler et al., 2004; Deforche et al., 2015). VanKim and Nelson (2013) found that students who engaged in frequent vigorous physical activity were less likely to report poor mental health and perceived stress than students who exercised less frequently.

Various theories provide explanations for the well-documented association between physical activity and improved physical and mental health. For example, the “fitness hypothesis” suggests that cardiovascular and sympathetic nervous systems show diminished stress responses after improvements in fitness level (Holmes and Roth, 1985), while the “mastery hypothesis” suggests that exercise gives an individual a sense of accomplishment and consequent improvement in mood (Carmack et al., 1999). It has also been suggested that physical activities can provide individuals with an effective method of distraction from stressful situations (Carmack et al., 1999). As such, the existing literature in this domain suggests that physical exercise may help individuals manage high levels of stress they are experiencing in collegiate settings.

### Stress and Mindfulness

More recent research has examined the role of mindfulness (e.g., Kabat-Zinn, 1982, 2003) in student adaptation. Mindfulness can be defined as “the awareness that emerges through paying attention on purpose, in the present moment, and non-judgmentally to the unfolding experience moment by moment” (Kabat-Zinn, 2003, p. 145). As such, mindfulness practice is an attention-related construct that focuses on improving one’s intention, attention, and attitude in order to gain greater awareness in non-judgmental and accepting ways (Shapiro et al., 2008). The overall goal of mindfulness practice is to be both present and compassionate in life’s moment-to-moment experiences (Shapiro et al., 2008). A growing body of research suggests that higher levels of mindfulness are associated with lower levels of perceived stress, anxiety symptoms, depressive symptoms, suicidal ideation, and pain (Baer, 2006; Araas, 2008; Anastasiades et al., 2017).

Research on college students has begun to elucidate associations between mindfulness and positive outcomes (e.g., reduction in stress and anxiety) among college students (for a review of this topic, see Bamber and Schneider, 2016). For example, Zimmaro et al. (2016) demonstrated that undergraduates with higher dispositional mindfulness perceived less stress and also exhibited lower cortisol levels, suggesting that mindfulness may be an effective mediator of physiological and psychological stress in a college setting. Similarly, Shapiro et al. (2008) reported that undergraduate students who increased mindfulness through a mindfulness-based stress reduction (MBSR) program (Kabat-Zinn, 1982, 2003) experienced reductions in perceived stress. Other similar studies have demonstrated that full MBSR programs are not necessary to achieve the desired effect; Call et al. (2014) found that brief, discrete elements of standard MBSR treatments are sufficient to significantly attenuate anxiety and stress in college students. A meta-analysis of cognitive, behavioral, and mindfulness interventions for college students suggested that mindfulness interventions can successfully reduce anxiety and depressive symptoms (Regehr et al., 2013). Similarly, Lynch et al. (2011, 2018) provide evidence to indicate the Mindfulness-Based Coping with University Life 8 week program significantly reduced negative mental health outcomes among students. The literature reviewed herein suggests that mindfulness is associated with adaptive functioning in college students and may be taught to reduce perceived stress and enhance well-being.

### The Current Study

The various lines of research discussed above provide clear evidence that college students can manage the perceived stress effectively through any or all of (a) adaptive coping, (b) physical activity, and (c) mindfulness. However, the differential contribution of each of these variables on student response to stress has not been studied, and thus comparisons of effectiveness or pragmatic interventional recommendations cannot differentiate among these coping methods. Therefore, this study considered all three variables, as well as potential confounding variables, as predictors of five aspects of student adjustment: perceived stress, loneliness, self-esteem, and symptoms of anxiety and depression. Although there are multiple indexes of adaptive functioning (e.g., academic achievement, social competence), we focus on self-esteem as it has both been widely studied and consistently found to be an indication of well-being (for a review on the topic, see Cast and Burke, 2002 see also Wagner et al., 2013; Randal et al., 2015). We focus on depression and anxiety as key outcome variables as prior theoretical and empirical research has suggested that these symptoms are core indices of the psychopathological process (e.g., Caspi et al., 2014) and are related to stress and coping (e.g., Skinner et al., 2003). Based on prior research, we predicted that greater use of adaptive coping, physical activity, and mindfulness would all be associated with student well-being. Given the limited intervention resources available, understanding the differential effects of coping approaches may guide both theoretical understanding of the underlying processes and pragmatic choices about interventions to further test. As comparisons between the three coping methods studied have not previously been made, and no clear theoretical reason exists to posit that one may be broadly more effective than another, no specific predictions were made with regard to magnitude of effect or comparisons between the three methods.

## Materials and Methods

### Procedure

Study procedures were approved by the Institutional Review Board of the first author’s institution. Data collection occurred at a small liberal arts college in New England over a 2-week period. All enrolled undergraduate students (*N* = 2496) received an email inviting them to participate in a study about their experiences with stress. A total of 1185 participants participated in the survey, representing a response rate of 47.48%. Emails contained a unique link to a Qualtrics^TM^ survey. This process was utilized to ensure participants only responded once. The survey took approximately 15 min to complete. Participants completed electronic consent, by selecting either, “I consent to participate” or “I do not consent to participate.” While participants’ responses to the survey were anonymized, participants were offered the opportunity to enter their email in a raffle to win one of ten $50 gift cards.

### Measures

#### Demographics

Participants were asked to self-report gender (male, female, or transgender), sexual orientation (heterosexual, gay/lesbian, bisexual, or other), race/ethnicity (African American, Asian Pacific Islander, White, Latino, mixed race, or other), as well as their perceived socioeconomic status (SES) (lower, middle, or upper).

#### Anxiety

The Generalized Anxiety Disorder-7 (GAD-7) scale was used to measure anxiety (Spitzer et al., 2006). Participants respond to seven items, indicating how often (0 = not at all to 3 = nearly every day) they are bothered by experiences such as “Feeling nervous, anxious or on edge” or “Being so restless that it is hard to sit still.” The items are summed to create a composite score; higher scores reflect greater levels of anxiety. For the GAD-7, the minimally clinically important difference (MCID), or the within-person change of clinical significance, is approximately four (Toussaint et al., 2020). In this study, the internal reliability was α = 0.92.

#### Depression

The Patient Health Questionnaire-9 (PHQ-9) was used to measure depressive symptomology (Kroenke et al., 2001). The PHQ-9 includes nine items that participants respond to as they consider how often they had been bothered by each of the items over the last 2 weeks. Items include “Little interest or pleasure in doing things” as well as “Trouble falling or staying asleep, or sleeping too much.” A four-point response scale is provided, which includes not at all (0), several days (1), more than half the days (2), and nearly every day (3). Summed items create a composite score; higher scores indicated greater levels of depressive symptoms. The MCID for the PHQ-9 is approximately five (Kroenke, 2012). The internal reliability was α = 0.88.

#### Loneliness

The UCLA Loneliness Scale (Vs. 3) was used to measure loneliness (Russell, 1996). Participants responded to 20 items, which included “How often do you feel you lack companionship” as well as “How often do you feel left out.” The response set included four responses, never (1), rarely (2), sometimes (3), and always (4). Higher scores reflect greater levels of loneliness. Internal reliability for this scale was α = 0.94.

#### Perceived Stress

The Perceived Stress Scale (PSS-10; Cohen et al., 1994) was used to measure participant stress levels. This 10-item self-report scale measures the degree to which participants appraise situations in their lives as stressful. Participants were asked to reflect on how they felt about certain situations in the last month, using a five-point Likert scale (0 = never; 4 = often). Example items include “In the last month, how often have you felt that you were on top of things?” as well as “In the last month, how often have you felt nervous and stressed?” Higher scores reflected greater levels of perceived stress. Internal reliability was α = 0.87.

#### Coping

The Brief COPE (Carver, 1997) assesses a variety of coping strategies used to manage life stressors. This 28-item self-report scale asks the participant to think of a stressful issue in his or her life and then answer the degree to which he or she used coping strategies, using a four-point Likert Scale ranging from 1 (I haven’t been doing this at all) to 4 (I’ve been doing this a lot). Fourteen subscales can be created to assess a variety of different coping behaviors, such as denial, behavioral disengagement, and active coping. Eight of these subscales are considered to be adaptive and six are considered to be maladaptive. Higher score indicates more frequent usage of a particular strategy. This study computed two aggregate coping score scores: engagement coping and disengagement coping. Each of these aggregate scores consisted of four subscales of the Brief COPE. Mean values for active coping, use of emotional support, use of instrumental support, and planning were used to calculate engagement coping; mean values of self-distraction, denial, substance use, and behavioral disengagement were used to calculate the disengagement coping subscale. The internal reliability was α = 0.89 for engagement coping and α = 0.76 for disengagement coping.

#### Self-Esteem

The 10-item Rosenberg Self-Esteem Scale (Rosenberg, 1965) was used to measure self-esteem. Participants responded to items utilizing a four-point scale, ranging from (1) strongly disagree to (4) strongly agree. The 10 items are summed, with higher scores indicating higher levels of self-esteem. Items included “I feel that I am a person of worth, at least on an equal basis with others” and “I certainly feel useless at times” (reversed coded). Internal reliability was α = 0.91.

#### Physical Activity

The Concise Physical Activity Questionnaire (CPAQ) was used to measure the amount and type of physical activity participants engaged in Sliter and Sliter (2014). The CPAQ is four-item self-report scale that asks how many days per week various types of physical activity were done for 20 consecutive minutes in the past month. Physical activities included light aerobics, moderate aerobics, vigorous aerobics, and muscle strengthening. Responses ranged from (0) physically unable to participate in an activity/elected to not participate in an activity, (1) 1 day per week or less, (2) 2–3 days per week, (3) 4–5 days per week, (4) 6–7 days per week. A composite score is computed by multiplying the responses to the vigorous aerobic activity by 2.5 and summing this score with the other three items. Internal reliability was α = 0.88.

#### Mindfulness

Two subscales from the Five Facet Mindfulness Questionnaire (Baer et al., 2006) were used to measure the mindfulness constructs of Acting with Awareness and Non-judging of Inner Experience. Each subscale contained eight items. Participants rated items on a five-point Likert scale (1 = never or rarely true; 5 = very often or always true). Items for the subscales included “When I do things my mind wanders off and I’m easily distracted” (Reverse coded – Acting with Awareness) and “I criticize myself for having irrational or inappropriate emotions” (Reverse coded, Non-Judging of Inner Experience). The three other subscales from the FFMQ were excluded because of evidence suggesting participant misinterpretation of questions (Describing; Jensen et al., 2016), and empirical evidence of performance inconsistent with the two included subscales used (Observing and Non-reactivity; Jensen et al., 2016; Eilenberg et al., 2017; Hedman et al., 2017; Rudkin et al., 2018). The internal reliability for the subscales used were α = 0.92 for Acting with Awareness and α = 0.96 for Non-Judging of Inner Experience.

### Data Preparation and Analysis

All analyses were performed using SPSS 25 (IBM Corp,, 2017). Basic descriptive statistics were calculated, including means and standard deviations of key variables as well as participant demographics. Participants were included in analyses if they completed the measures used in that analysis; thus, there is minor variability in *n* between each analysis.

Statistical assumptions of hierarchical regressions were tested prior to further analysis. Correlations between variables indicated that none of the variables were overly correlated with each other. Collinearity statistics were also examined to determine acceptable levels of Tolerance and VIF were met. Four hierarchical linear regression models were run, separately predicting Stress (as measured by the PSS; Cohen et al., 1994), Anxiety (as measured by the GAD-7; Spitzer et al., 2006), Depression (as measured by the PHQ-9; Kroenke et al., 2001), and Loneliness (as measured by the UCLA Loneliness Scale; Russell, 1996). In each model, gender (coded as male = 0 and female = 1), race (coded as Caucasian = 0 and all other = 1), and SES (coded as 0 = low and middle SES and 1 = high SES) were entered in step 1. Step 2 added engagement and disengagement coping (as measured by the Brief COPE; Carver, 1997; see above for discussion of the creation of engagement and disengagement grouped scales), physical activity (as measured by the CPAQ; Sliter and Sliter, 2014), and mindful awareness and mindful non-judgment (as measured by the Five Facet Mindfulness Questionnaire; Baer et al., 2006; see above for discussion of the selection of the two subscales used). Models were assessed for overall model prediction of variability for the outcome measure.

## Results

Demographics are reported in Table 1. A series of correlation analyses between the predictor variables and the dependent variables are presented in Table 2. The regression model predicting stress scores was statistically significant, *F*(8,889) = 119.33, *p* < 0.001, adjusted *R*^2^ = 0.51. Disengagement coping (β = 0.26, *p* < 0.001), mindfulness acting with awareness (β = −0.24, *p* < 0.001), and mindfulness non-judgment (β = −0.34, *p* < 0.001) all emerged as significant predictors for stress. The regression model predicting anxiety was also statistically significant, *F*(8,887) = 100.83, adjusted *R*^2^ = 0.47; disengagement coping (β = 0.24, *p* < 0.001), engagement coping (β = 0.06, *p* < 0.05), mindfulness acting with awareness (β = −0.19., *p* < 0.001), and mindfulness non-judgment (β = −0.36, *p* < 0.001) all emerged as significant predictors.

**TABLE 1:** Demographic characteristics of study participants.

Characteristic	*n*	%
**Current class standing**		
First Year	273	23.0
Sophomore	300	25.3
Junior	222	18.7
Senior	390	32.9
**Race/Ethnicity**		
African American	40	3.4
API	127	10.7
Latino	69	5.8
White	837	70.6
Mixed Race/Other	112	9.5
**Gender**		
Male	508	43.0
Female	674	57.0
Transgender	3	<0.1
**Sexual orientation**		
Heterosexual/Straight	1013	85.5
Gay/Lesbian	55	4.6
Bisexual	92	7.8
Other	25	2.1
**Perceived family socioeconomic status**		
Lower	132	11.1
Middle	594	50.1
Upper	459	38.7

**TABLE 2:** Summary of intercorrelations, means, and standard deviations for mental health outcomes and predictors.

Measures	1	2	3	4	5	6	7	8	9	10	M	SD
1. Stress		0.72***	0.67***	0.51***	−0.61***	0.54***	0.18***	−0.10***	−0.54***	−0.62***	18.16	6.44
2. Anxiety			0.71***	0.50***	−0.56***	0.52***	0.23***	−0.07*	−0.50***	−0.61***	14.84	5.70
3. Depression				0.60***	−0.62***	0.63***	0.12***	−0.14***	−0.56***	−0.61***	16.70	5.68
4. Loneliness					−0.54***	0.42***	–0.06	−0.20***	−0.37***	−0.47***	43.35	11.86
5. Self-esteem						−0.47***	–0.06	0.17***	0.47***	0.63***	29.95	5.96
6. Disengaged coping							0.22***	–0.05	−0.42***	−0.49***	3.47	1.05
7. Engaged coping								–0.01	−0.10**	−0.22***	4.92	1.46
8. CPAQ									0.08*	0.08*	23.54	9.93
9. Mindfulness awareness										0.51***	24.93	6.94
10. Mindfulness non-judgment											26.68	8.96

*p < 0.05, **p < 0.01, ***p < 0.001. CPAQ, Concise Physical Activity Questionnaire.

The overall regression model predicting depression was statistically significant, *F*(8,888) = 149.19, adjusted *R*^2^ = 0.57. The following four predictors were significant: disengagement coping (β = 0.40, *p* < 0.001), engagement coping (β = −0.06, *p* < 0.001), physical activity (β = −0.05), mindfulness acting with awareness (β = −0.24, *p* < 0.001), and mindfulness non-judgment (β = −0.28, *p* < 0.001). The final regression model for loneliness was significant, *F*(8,889) = 58.31, adjusted *R*^2^ = 0.34, and in this model, all five predictor variables were significant (disengagement coping: β = 0.26, *p* < 0.001; engagement coping: β = −0.20, *p* < 0.001; physical activity: β = −0.13, *p* < 0.001; mindfulness acting with awareness: β = −0.11, *p* < 0.001; mindfulness non-judgment: β = −0.29, *p* < 0.001). The regression model predicting self-esteem was statistically significant, *F*(8,888) = 103.76, *p* < 0.001, adjusted *R*^2^ = 0.48 and all five predictors were significant as well (disengagement coping: β = −0.19, *p* < 0.001; engagement coping: β = 0.11, *p* < 0.001; physical activity: β = −0.09, *p* < 0.001; mindfulness acting with awareness: β = 0.13, *p* < 0.001; mindfulness non-judgment: β = 0.47, *p* < 0.001). Regression analyses are summarized in Tables 3, 4.

**TABLE 3:** Hierarchal regression analyses predicting stress, anxiety, and depression.

	Model 1 Predicting Stress						Model 2 Predicting Anxiety						Model 3 Predicting Depression					
Variable	*B*	*SE B*	β	*t*	*R*^2^	Δ*R*^2^	*B*	*SE B*	β	*t*	*R*^2^	Δ*R*^2^	*B*	*SE B*	β	*t*	*R*^2^	Δ*R*^2^
Step 1					0.06						0.05						0.04	
Gender	–2.38	0.43	–0.18	−5.55***			–2.05	0.38	–0.17	−5.39***			–1.05	0.37	–0.09	−2.81**		
Race	0.88	0.47	0.06	1.87			0.63	0.42	0.05	1.50			1.23	0.41	0.10	3.01**		
SES	–1.81	0.44	–0.14	−4.10***			–1.21	0.39	–0.10	−3.08**			–1.63	0.38	–0.14	−4.26***		
Step 2					0.51	0.46					0.47	0.43					0.57	0.52
Disengagement coping	1.59	0.17	0.26	9.21***			1.27	0.16	0.24	8.15***			2.13	0.14	0.40	15.19***		
Engagement coping	–0.07	0.11	–0.02	0.60			0.22	0.10	0.06	2.26*			–0.24	0.09	–0.06	−2.71**		
CPAQ	–0.01	0.02	–0.02	–0.74			0.01	0.02	0.01	0.34			–0.03	0.01	–0.05	−1.98*		
Mindfulness awareness	–0.22	0.03	–0.24	−8.48***			–0.16	0.02	–0.19	−6.62***			–0.19	0.02	–0.24	−9.02***		
Mindfulness non-judgment	–0.24	0.02	–0.34	−11.38***			–0.23	0.02	–0.36	−11.60***			–0.17	0.02	–0.28	−9.81***		
																		

*p < 0.05, **p < 0.01, ***p < 0.001. *R*^2^ values are adjusted; Gender was coded 0 = female, 1 = male; race was coded Caucasian = 0 and Non-Caucasian = 1; SES was coded 0 = low and middle SES and 1 = high SES. Mindfulness Awareness, Mindfulness acting with awareness; CPAQ, Concise Physical Activity Questionnaire.

**TABLE 4:** Hierarchal regression analyses predicting loneliness and self-esteem.

	Model 4 Predicting Loneliness						Model 5 Predicting Self-esteem					
Variable	*B*	*SE B*	β	*t*	*R*^2^	Δ*R*^2^	*B*	*SE B*	β	*t*	*R*^2^	Δ*R*^2^
Step 1					0.04						0.05	
Gender	−1.65	0.79	0.07	−2.08*			−1.65	0.79	0.07	−2.08*		
Race	3.28	0.87	0.13	3.75*			3.28	0.87	0.13	3.75*		
SES	−3.14	0.82	0.13	−3.84**			−3.14	0.82	0.13	−3.84**		
Step 2					0.34	0.19					0.48	0.42
Disengagement coping	2.90	0.37	0.26	7.84***			−1.10	0.16	−0.19	−6.52***		
Engagement coping	−1.61	0.23	−0.20	−6.97***			0.45	0.10	0.11	4.37***		
CPAQ	−0.15	0.03	−0.13	−4.48***			0.06	0.02	0.09	3.66***		
Mindfulness awareness	−0.18	0.06	−0.11	−3.32***			0.11	0.03	0.13	4.64***		
Mindfulness non-judgment	−0.39	0.05	−0.29	−8.42***			0.31	0.02	0.47	15.15***		

*p < 0.05, **p < 0.01, ***p < 0.001. *R*^2^ values are adjusted; Gender was coded 0 = female, 1 = male; race was coded Caucasian = 0 and Non-Caucasian = 1; SES was coded 0 = low and middle SES and 1 = high SES. Mindfulness Awareness, Mindfulness acting with awareness; CPAQ, Concise Physical Activity Questionnaire.

## Discussion

College students are experiencing high levels of stress (Bayram and Bilgel, 2008; Leary and DeRosier, 2012). After transitioning to college, students report increased difficulty in several domains (Conley et al., 2014), including mental health symptoms and considerable substance use (e.g., Johnston et al., 2016). Such research findings have highlighted the need to better understand different approaches for the management of stress in college students. To our knowledge, no study to date has examined the differential contribution of three stress management approaches – coping, physical activity, and aspects of mindfulness – on college student adjustment/maladjustment.

Participants in this study experienced high levels of stress. According to available cutoff scores for the Perceived Stress Scale (PSS), on average, students endorsed experiencing “moderate” levels of stress; roughly 10% of the sample reported experiencing “high” levels of stress. Further, on average, students self-reported experiencing “severe” levels of anxiety and “moderately severe” levels of depression. This suggests that participants in this study experienced both high levels of stress and adjustment difficulties, as evidenced by elevated rates of anxiety and depressive symptoms.

Hierarchical linear regression results supported the view that coping, physical activity, and components of mindfulness are all predictive of student adaptation. Engagement coping was associated with lower levels of depression and loneliness as well as with higher levels of self-esteem. Conversely, greater levels of disengagement coping were associated with greater levels of perceived stress, anxiety, depression, and loneliness as well as with lower levels of self-esteem. These results align with prior research documenting that engagement coping is generally associated with adaptation while disengagement coping is generally associated with maladaptation (Clarke, 2006; Carver and Connor-Smith, 2010; Mahmoud et al., 2012). Unexpectedly, engagement coping was predictive of higher levels of anxiety in this study. Although inconsistent with the implicit directionality of the regression model tested, it is possible that students who are experiencing high levels of anxiety are reporting the use of more coping strategies, even those that are conceptualized as being adaptive. Unfortunately, our methods did not allow us to differentiate between prevalence and effectiveness of coping strategy use. Future longitudinal or interventional research should evaluate the nature of this relationship.

Physical activity emerged as a significant predictor of three outcome variables in hierarchical regression analyses. Consistent with prior research and our hypothesis, higher rates of physical activity were predictive of lower levels of depression, lower levels of loneliness, and higher levels of self-esteem. Prior research has documented that physical activity is associated with a reduction in stress and anxiety while also elevating mood (Dunn et al., 2001; Penedo and Dahn, 2005; Galper et al., 2006; Baghurst and Kelley, 2014). Prior research has also documented the benefits of physical activity on college student well-being and functioning (VanKim and Nelson, 2013; Wunsch et al., 2017). Our results directly align with the prior research by documenting that physical activity is a significant predictor for three indices of student well-being: depression, loneliness, and self-esteem.

This study found that the two indices of mindfulness – mindful awareness and mindful non-judgment – were both associated with lower stress, lower anxiety, lower depression, lower loneliness, and higher self-esteem. Examination of beta weights suggested that the magnitude of this effect was particularly strong for both mindfulness variables *relative to* other predictor variables. The finding that mindful awareness and mindful non-judgment emerged as strong predictors within statistical models that also considered coping and physical activity represents a notable finding of this study. Such statistics suggest that components of mindfulness are closely linked to college student adjustment – a view that is also supported by prior research in the field. Existing research has documented that higher levels of mindfulness are linked to lower levels of perceived stress, anxiety symptoms, depression symptoms, and suicidal ideation (Baer et al., 2006; Anastasiades et al., 2017). Further, recent research has documented that mindfulness attributes in college students are associated with a wide range of positive outcomes (e.g., Bamber and Schneider, 2016; Zimmaro et al., 2016).

Results from this study have implications for applied work. Given that all examined stress management approaches were predictive of student adaptation, students could be educated about the potential benefits of being physically active, using adaptive coping behaviors (e.g., engaging with the source of stress), reducing the use of disengaged coping strategies and fostering mindfulness. Even while considering other adaptive stress responses, mindfulness emerged as a particularly consistent and strong predictor of well-being. Given that mindfulness skills can be taught to emerging adult populations to enhance well-being (Shapiro et al., 2008; Regehr et al., 2013; Call et al., 2014), our results support the view that mindfulness skills could be taught to college students as a prevention and intervention for adjustment problems.

### Limitations

The cross-sectional nature of the data collected does not allow for causal inferences. As such, our findings cannot speak to whether the examined stress management strategies (coping, physical activity, and mindfulness) *caused* the outcomes of interest, perceived stress, anxiety symptoms, depression symptoms, loneliness, and self-esteem. It is possible that these results reflect a relationship between stress and mindfulness that is either bidirectional or points in the opposite direction described, such that stress reduces mindfulness (which may then in turn increase stress). More complex dynamics still allows for intervention at either point in the causal cycle, but the present study cannot distinguish between those possibilities. Further, the narrow range of participant ages restricted our ability to assess changes across college career. Finally, this study recruited participants from a small and competitive liberal arts college in New England, which may make it difficult to generalize our findings to the overall population of college students. Future research on this topic could address these limitations by using a longitudinal or experimental approach and could recruit participants that are representative of the overall college (or perhaps emerging adult) populations.

## Conclusion

These findings shed light on the differential contributions of coping, physical activity, and aspects of mindfulness on college student functioning. Our findings suggest that students who report higher levels of mindfulness appear to be better adjusted across several domains of functioning. As such, efforts to foster mindfulness in college students may support well-being and protect emerging adults from the potentially harmful effects of stress.

## Data Availability Statement

The datasets generated for this study will not be made publicly available to protect the confidentiality of research participants the data is not publicly available.

## Ethics Statement

The studies involving human participants were reviewed and approved by the Middlebury College Institutional Review Board. The participants provided their electronic informed consent to participate in this study.

## Author Contributions

RM, MS, JS, EL, TR, and CR contributed to the design and data collection. RM, MS, JS, and VP contributed to analyses. All authors contributed to the writing of drafts of the manuscript. RM, MS, and VP completed final manuscript for submission.

## Conflict of Interest

The authors declare that the research was conducted in the absence of any commercial or financial relationships that could be construed as a potential conflict of interest.
